# Deciphering SARS CoV-2-associated pathways from RNA sequencing data of COVID-19-infected A549 cells and potential therapeutics using in silico methods

**DOI:** 10.1097/MD.0000000000029554

**Published:** 2022-09-02

**Authors:** Peter Natesan Pushparaj, Laila Abdullah Damiati, Iuliana Denetiu, Sherin Bakhashab, Muhammad Asif, Abrar Hussain, Sagheer Ahmed, Mohammad Hamid Hamdard, Mahmood Rasool

**Affiliations:** a Center of Excellence in Genomic Medicine Research, Department of Medical Laboratory Technology Faculty of Applied Medical Sciences, King Abdulaziz University, Jeddah, Saudi Arabia; b Centre for Transdisciplinary Research, Department of Pharmacology, Saveetha Dental College and Hospitals, Saveetha Institute of Medical and Technical Sciences, Chennai, India; c Department of Biology, College of Science, University of Jeddah, Jeddah, Saudi Arabia; d King Fahad Medical Research Center, Faculty of Applied Medical Sciences, King Abdulaziz University, Jeddah, Saudi Arabia; e Department of Biochemistry, Faculty of Biological Sciences, King Abdulaziz University, Jeddah, Saudi Arabia; f Department of Biotechnology, BUITEMS, Quetta, Pakistan; g Office of Research Innovation and Commercialization, BUITEMS, Quetta, Pakistan; h Shifa College of Pharmaceutical Sciences, Shifa Tameer-e-Millat University Islamabad, Pakistan; i Faculty of Biology, Kabul University, Kabul, Afghanistan.

**Keywords:** A549 cells, BioJupies, COVID-19, in silico, iPathwayGuide, natural products, next-generation knowledge discovery, RNA Seq, synthetic drugs

## Abstract

Coronavirus (CoV) disease (COVID-19) identified in Wuhan, China, in 2019, is mainly characterized by atypical pneumonia and severe acute respiratory syndrome (SARS) and is caused by SARS CoV-2, which belongs to the Coronaviridae family. Determining the underlying disease mechanisms is central to the identification and development of COVID-19-specific drugs for effective treatment and prevention of human-to-human transmission, disease complications, and deaths.

**Methods:**

Here, next-generation RNA sequencing (RNA Seq) data were obtained using Illumina Next Seq 500 from SARS CoV-infected A549 cells and mock-treated A549 cells from the Gene Expression Omnibus (GEO) (GSE147507), and quality control (QC) was assessed before RNA Seq analysis using CLC Genomics Workbench 20.0. Differentially expressed genes (DEGs) were imported into BioJupies to decipher COVID-19 induced signaling pathways and small molecules derived from chemical synthesis or natural sources to mimic or reverse COVID -19 specific gene signatures. In addition, iPathwayGuide was used to identify COVID-19-specific signaling pathways, as well as drugs and natural products with anti-COVID-19 potential.

**Results:**

Here, we identified the potential activation of upstream regulators such as signal transducer and activator of transcription 2 (STAT2), interferon regulatory factor 9 (IRF9), and interferon beta (IFNβ), interleukin-1 beta (IL-1β), and interferon regulatory factor 3 (IRF3). COVID-19 infection activated key infectious disease-specific immune-related signaling pathways such as influenza A, viral protein interaction with cytokine and cytokine receptors, measles, Epstein-Barr virus infection, and IL-17 signaling pathway. Besides, we identified drugs such as prednisolone, methylprednisolone, diclofenac, compound JQ1, and natural products such as Withaferin-A and JinFuKang as candidates for further experimental validation of COVID-19 therapy.

**Conclusions:**

In conclusion, we have used the in silico next-generation knowledge discovery (NGKD) methods to discover COVID-19-associated pathways and specific therapeutics that have the potential to ameliorate the disease pathologies associated with COVID-19.

## 1. Introduction

**Figure 1.**
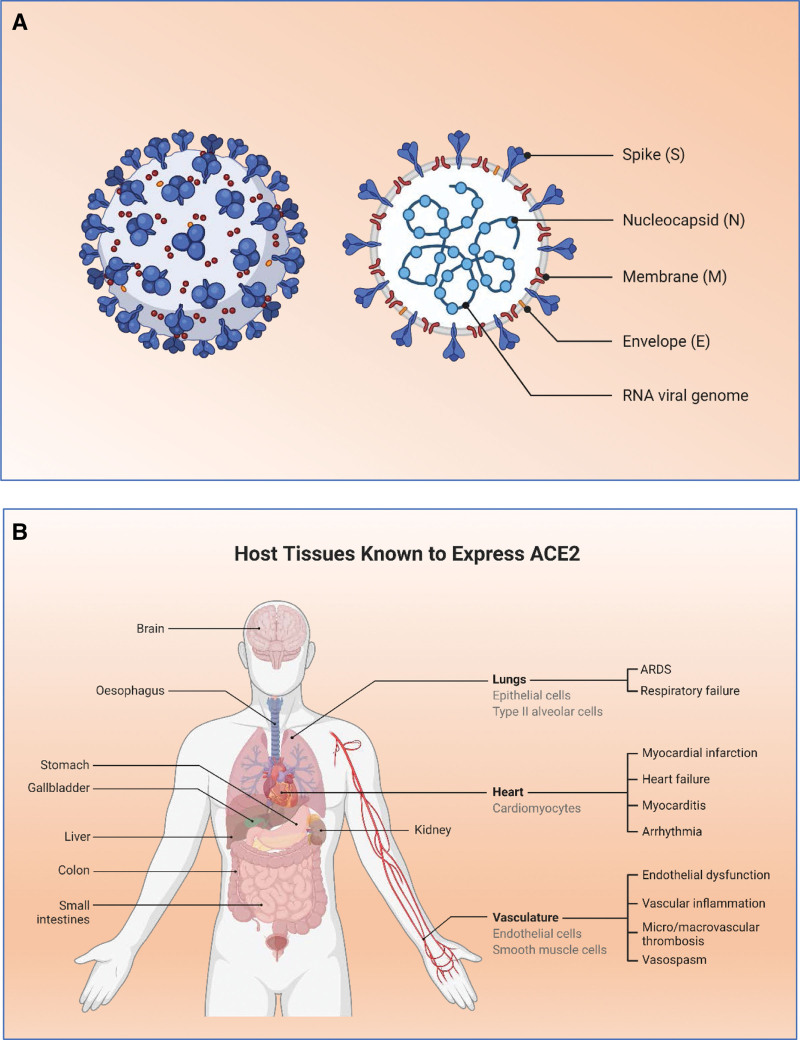
Structure of SARS-CoV 2. (A) The structure depicted based on electron microscopic observations of coronavirus showing the surface protein particles S, N, M, and E and shows a corona-like shape. (B) The host tissues expressing ACE2 receptors. (C) The mechanism of entry of SARS CoV2 into the host cells (this figure was created using the graphic tools offered by BioRender.com with an academic license).

Coronavirus disease 2019 (COVID-19) is caused by a type of coronavirus (CoV), severe acute respiratory syndrome (SARS) virus 2 (SARS CoV-2). COVID-19 is characterized by symptoms ranging from a mild cold to more severe illnesses, such as SARS, sudden stroke, gastrointestinal complications, and multiple organ failure, even leading to death in some patients.^[1–3]^ Coronaviruses belong to the Coronaviridae family, and the presence of viral spike proteins in the virus gives it a halo or corona-like appearance under the electron microscope (Fig. 1A). A novel coronavirus (nCoV) was discovered in Wuhan, China in 2019 as the cause of a human respiratory outbreak that resulted in severe atypical pneumonia.^[4,5]^ and is the source of the current global pandemic affecting all levels of society.^[6]^

The World Health Organization (WHO) has renamed this nCoV as SARS-CoV 2, which is the causative agent of COVID-19.^[4,5,7]^ COVID-19 is highly transmissible and pathogenic compared with other viral infections, and the exact mortality rate has yet to be determined because the pandemic is not yet under control in several countries, resulting in unprecedented protective measures, partial or complete lockdowns, travel restrictions, etc.^[8]^ As of March 7, 2022, COVID-19 had already infected more than 446 million people in 195 countries and territories around the world and killed approximately 6 million people, according to data from the Johns Hopkins Coronavirus Dashboard.^[9]^ However, the exact mortality rate will not be calculated or determined until the COVID-19 epidemic reaches a plateau. The United States of America and WHO have declared the SARS-CoV-2 outbreak a public health emergency because it is more contagious than the severe acute respiratory syndrome coronavirus (SARS-CoV) and Middle East respiratory syndrome coronavirus (MERS-CoV).^[5,8,9]^ SARS-CoV-2 possesses a nucleocapsid with a positive-sense RNA genome. Host cells express SARS-CoV-2 nucleoproteins and the nucleocapsid protein (N protein), which is the most abundant, highly immunogenic protein, and is required for CoV RNA synthesis. The N protein is a structural protein that binds to the CoV RNA genome and forms a capsid around viral RNA. However, the spike protein (S protein) is critical for binding between SARS CoV-2 and angiotensin-converting enzyme 2 (ACE2) surface receptors on host cells (Fig. 1B), thus facilitating coronavirus entry into host cells.,^[10]^ respectively (Fig. 1C).

Although COVID-19 vaccines are currently available as preventive measures and many are in the research and development phase,^[11,12]^ deciphering the underlying pathological mechanisms is central to identifying and developing COVID-19 specific drugs to effectively treat and prevent human-to-human transmission, COVID-19 complications, and deaths. In silico methodologies can be successfully used to identify potential drugs and natural products based on high-dimensional RNA-seq datasets derived from various disease pathologies.^[13,14]^ We have recently shown that the next-generation knowledge discovery (NGKD) platforms can effectively be used to uncover the gene signatures regulated by COVID-19 and the potential therapeutics using RNAseq datasets derived from normal human primary bronchial epithelial (NHBE) cells.^[13]^ However, in the present study, the raw RNA Seq reads (single-end) (FASTQ files) in quadruplicate obtained from SARS CoV-infected A549 cells and mock-treated A549 cells using Illumina Next Seq 500 were obtained from the Gene Expression Omnibus (GEO) (accession number: GSE147507) and quality control (QC) was evaluated before RNA Seq analysis using CLC Genomics Workbench 20.0 (Qiagen, USA). After the initial QC, the RNA Seq reads were imported into the CLC Genomics Workbench 20.0 (Qiagen, USA) before RNA Seq analysis and evaluated using NGKD platforms such as BioJupies^[15]^ and iPathwayGuide (Advaita Bioinformatics, USA) to decipher the disease-specific molecular signatures and a series of small molecules derived from either synthetic or natural sources to mimic or reverse the COVID-19 gene signatures.

## 2. Materials and Methods

### 2.1. Ethical statement

Animal models and human subjects were not used in this study. This study was performed using RNA-seq datasets from next-generation sequencing experiments with A549 cells. The raw data were obtained from the Gene Expression Omnibus (GEO), as indicated in the Data Source section below. Therefore, they were exempt from institutional review board (IRB) approval.^[13,14]^

### 2.2. Next-Generation Sequencing (NGS) data source

Raw RNA Seq reads (single-end) (FASTQ format) in quadruplicate obtained with Illumina Next Seq 500 from A549 cells infected with SARS CoV-2 and mock-treated A549 cells were obtained from the Gene Expression Omnibus (GEO) (accession number: GSE147507)^[16]^ and were used for subsequent downstream analysis with high-throughput NGKD platforms.

### 2.3. COVID-19 RNA Seq data from A549 cells – quality control

Raw RNA Seq reads (single-end) in quadruplicates (FASTQ files) derived from SARS CoV-infected A549 cells and mock-treated A549 cells using Illumina Next Seq 500 were derived from the GEO, and quality control (QC) was evaluated using CLC Genomics Workbench 20.0 (Qiagen)^[13]^ to obtain the differentially expressed genes (DEGs) before RNA Seq analysis.

### 2.4. COVID-19 RNA Seq data from A549 cells – differential gene and transcript expression analysis

RNA Seq reads were imported into CLC Genomics Workbench 20.0 (Qiagen) after the QC step. The RNA Seq Analysis Tool in the Biomedical Genomics Analysis plugin of the CLC Genomics Workbench was used to extract all annotated transcripts using both Homo sapiens (hg38) _genes (Gene track) and Homo sapiens (hg38) _mRNA (mRNA track) and mapped to the human reference genome (GRCh38). A gene expression track (GE) was generated for A549 cells infected with SARS CoV-2 and corresponding mock reads (test vs. control). In addition, the differential expression tool was used in the two groups in the CLC Genomics Workbench to perform a statistical test for differential expression for a set of expression tracks (test vs. control). Here, a multifactorial statistic based on a negative binomial generalized linear model (GLM) is used, and the differential expression in the two groups tool deals with one factor and two groups. In this analysis, “Total Exon Read” values were used for GE. Differentially expressed genes (DEGs) were generated for the test compared to the corresponding control and used for further downstream analysis using the NGKD platform.

### 2.5. BioJupies analysis of RNASeq data

BioJupies was used to analyze the DEGs generated using the CLC Genomics Workbench to identify novel signaling pathways, disease-specific gene networks, and a range of drugs and small molecules derived from natural sources to mimic or reverse disease-specific gene signatures.^[15]^ In Biojupies, RNASeq datasets were compressed into an HDF5 data package and uploaded to Google Cloud. Raw data were normalized to log10 counts per million (log CPM) and differentially expressed genes between the control and experimental groups were determined using the R package limma.^[17]^ The principal component analysis function in the Python module of sklearn was used to transform log CPM based on the Z-score method to generate the PCA plot, and Clustergrammer^[18]^ was used to generate interactive heat maps, and the DEGs were then sent to Enrichr.^[19]^ In the volcano plot, DEGs were plotted on the x-axis and *P* values were corrected using the Benjamini-Hochberg method, transformed (−log10), and plotted on the y-axis.^[20,21]^ However, average gene expression is shown on the x-axis in the MA plot, and *P* values were corrected, transformed (−log10), and plotted on the y-axis using the Benjamini-Hochberg method.^[20,21]^ Gene Ontology (GO) and pathway enrichment analyses were performed with both upregulated and downregulated genes in Enrichr. Significant GO terms and pathways (KEGG, WiKiPathways, and Reactome) were calculated using a cut-off value of < 0.1 after applying the Benjamini–Hochberg correction.^[20,21]^

### 2.6. L1000CDS2 and L1000FWD analyses

The L1000CDS2 analysis was performed by submitting the best 2000 DEGs to the L1000CDS2 signature search API.^[22]^ Similarly, the L1000FWD analysis was performed by submitting the top 2000 DEGs to the L1000FWD signature search API.^[23]^

### 2.7. In silico analysis of RNASeq expression data using iPathwayGuide

The impact analysis method (IAM)^[24]^ in the iPathwayGuide was used to determine the differentially regulated signaling pathways, gene ontologies, and upstream drugs or natural products, as previously described.^[13]^ Briefly, the pathway score was calculated based on the p-value obtained using Fisher’s method. The *P* value was corrected using multiple testing corrections for the false discovery rate (FDR) and Bonferroni correction.^[25,26]^ The FDR has significant power, but it controls only the family-based false-positive rate.^[20,21]^ Pathways and gene interactions with DEGs were generated using the KEGG database.^[27]^ For each Gene Ontology term (GO), the number of DEGs annotated with the term was compared with the randomly expected DEGs.^[28,29]^ iPathwayGuide used an overrepresentation approach to calculate the statistical significance of observing at least the specified number of DEGs.^[30–32]^ The hypergeometric distribution was used to calculate *P* values in the iPathwayGuide analysis and corrected for multiple comparisons using FDR and Bonferroni.^[30–32]^

### 2.8. Prediction of upstream drugs or natural products with iPathwayGuide

The prediction of upstream chemicals, drugs, and toxicants (CDTs) was based on two types of information: (i) the enrichment of DEGs from experiments and (ii) a network of interactions from the Advaita Knowledge Base (AKB v2012).^[30–32]^

### 2.9. Upstream CDTs predicted to be present (or overabundant)

The research hypothesis refers to the presence of CDT. This hypothesis is useful for investigating whether a given phenotype is influenced by the presence of a particular chemical, drug, or toxicant.^[30–32]^ For each CDT u, the number of consistent DE genes after u, DTA(u), is compared to the number of measured target genes expected to be both consistent and DE. iPathwayGuide uses an over-representation approach to calculate the statistical significance of observing at least a given number of consistent DE genes. The *P* value Ppres was calculated using a hypergeometric distribution.^[30–32]^ The analysis uses Fisher’s standard method to combine the *P* values into a test statistic.^[33]^

### 2.10. Upstream CDTs predicted to be absent (or insufficient)

In parallel with the upstream CDTs predicted to be present, Pabs and Pz were used to predict upstream CDTs that were absent. This hypothesis is important when investigating whether a given phenotype is affected by the absence of a particular chemical necessary for the proper functioning of the organism or cell. The research hypothesis states that the upstream CDT is insufficient under the conditions under study. For each upstream CDT u, the number of consistent DE genes downstream of u, DTI(u), was compared to the number of measured target genes expected to be both consistent and DE by chance. Using Fisher’s method, the analysis combines Pabs and Pz, with Pz considered only for significantly negative z-scores (z ≤ −2).^[30–32]^

### 2.11. Swiss target prediction of potential anti- COVID-19 compounds

The isomeric simplified molecular-input line-entry system (SMILES) codes of prednisolone and withaferin-A were used in the SwissTarget Prediction tool to identify protein targets.^[34,35]^ Ligand-based target prediction for both prednisolone and withaferin-A was performed as previously described.^[35,36]^

### 2.12. The Open Targets Platform analysis of anti-COVID-19 compounds

The Open Targets Platform web tool was used to uncover the molecular targets of prednisolone and withaferin-A associated with COVID-19 disease pathology (date accessed: January 12, 2020)^[35,37,38]^ The Open Targets Platform uses scientific evidence to assess and rank associations between targets and disease and to help prioritize targets.^[38]^ The query list of approximately 100 candidate molecular targets of prednisolone and withaferin-A was used to discover protein targets significantly (*P* < .05) associated with COVID-19.

## 3. Results

The present study was done using RNA-seq datasets obtained from next-generation sequencing experiments with mock-treated and SARS CoV-2 infected A549 cells.^[16]^ The raw RNA Seq reads (Single-End) (*FASTQ files*) in quadruplicates derived using Illumina Next Seq 500 from SARS CoV-infectedA549 cells, and mock-treated A549 cells were obtained from the Gene Expression Omnibus (GEO) (GSE147507), and quality control (QC) was evaluated before RNA Seq analysis using the CLC Genomics Workbench 20.0 (Qiagen). The DEGs were further analyzed using BioJupies and iPathwayGuide (Advaita Bioinformatics, USA) to decipher disease-specific signatures and an array of drugs and small molecules derived from natural sources to mimic or reverse disease-specific gene signatures.

**Figure 2.**
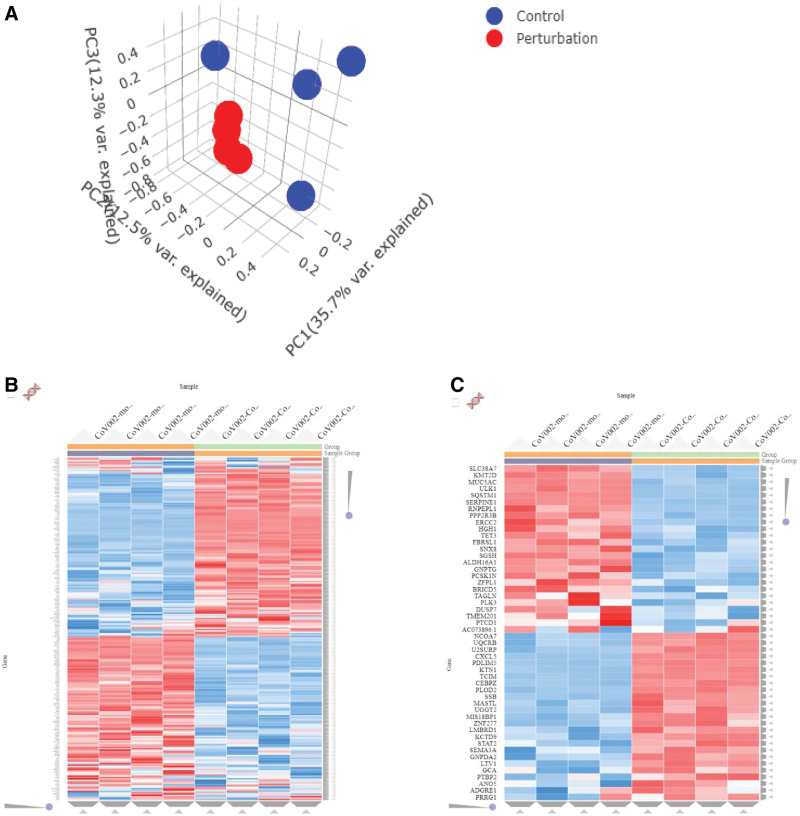
(A) Principal component analysis (PCA) was applied to identify global patterns in high-dimensional RNASeq datasets. (B) The heatmaps were generated top 250 DEGs and (C) the top 50 DEGs using Clustergrammer web tool for visualizing and analyzing high-dimensional RNASeq data. (D) Volcano plot was generated using transformed gene fold changes using log2 and displayed on the x-axis. (E) MA plot was based on average gene expression which was calculated using mean of the normalized gene expression values and displayed on the x-axis.

The global patterns in the high-dimensional RNA-seq datasets were uncovered using PCA analysis (Fig. 2A). The Clustergrammer web tool was used to generate interactive heatmaps for visualization and in-depth analysis of DEGs derived from high-dimensional RNASeq data of SARS CoV-infected A549 cells and mock-treated A549 cells (Fig. 2A–C). A volcano plot was generated using transformed gene fold changes using log2 and is shown on the x-axis (Fig. 2D). The MA plot was based on the average gene expression, which was calculated using the mean of the normalized gene expression values and is shown on the x-axis (Fig. 2E).

**Figure 3.**
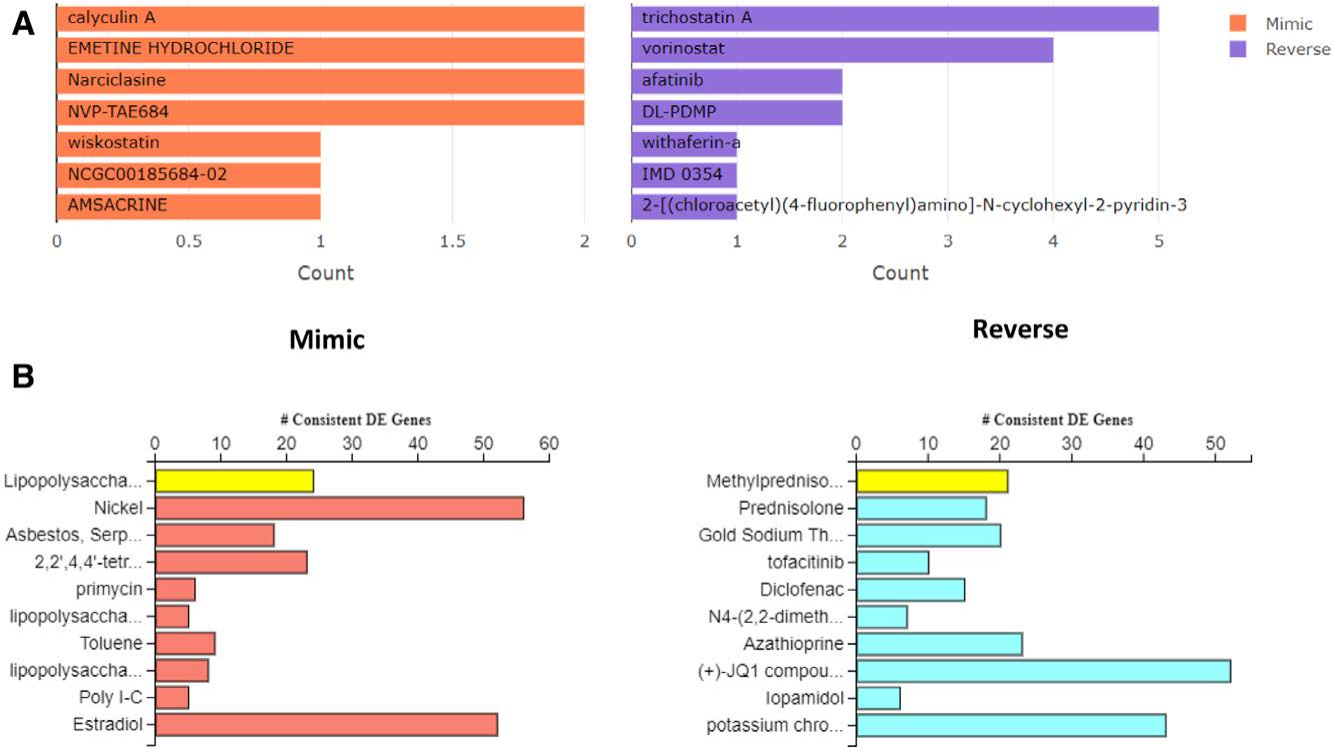
(A) The bar chart displaying the top small molecules identified by the L1000CDS2 query. The left panel displays the small molecules which mimic the observed gene expression signature, while the right panel displays the small molecules which reverse it. (B) Bar graphs show the synthetic drugs and natural compounds with similar (mimic) and opposite (reverse) molecular signatures based on iPathwayGuide analysis.

The bar chart (Fig. 3A) shows the top small molecules identified by the L1000CDS2 query. The left panel displays small molecules such as calyculin A, emetine hydrochloride, narliclasine, NVP-TAE684, wiskostatin, NCGC00185684-02, and amsacrine, which mimic the observed gene expression signature, while the right panel displays small molecules such as trichostatin A, vorinostat, afatinib, DL-PDMP, withaferin-A, IMD 0354, and 2-[(chloroacetyl) (4-fluorophenyl] amino-N-cyclohexyl-2 pyridine 3, which reverse it. In addition, natural products and drugs with opposite (Table 1) and similar molecular signatures (Table 2) based on the L1000FWD tool, which contains gene signatures from an array of human cell lines administered with more than 20,000 drugs and natural products. Withaferin-A, an active ingredient of the medicinal plant (Fig. 3A), *Withania somnifera* was found to reverse the COVID-19 induced molecular signatures in both L1000CDS2 and L1000FWD analyses along with other small molecule drugs.

**Table 1:** Natural products and drugs with opposite and similar molecular signatures based on L1000FWD web-based tool.

Opposite molecular signatures						
Signature ID	Drugs or natural products	Similarity score	*P* value	*q* value	Z-score	Combined score
CPC019_VCAP_24H:BRD-K50234570-001-06-6:10	EMF-bca1-16	**−0.0569**	**2.03E-10**	**8.67E-07**	**1.67**	**−16.15**
ERG005_VCAP_6H:BRD-K88378636-001-02-8:20	Withaferin-a	**−0.0544**	**1.54E-09**	**2.74E-06**	**1.65**	**−14.57**
CPC006_HCC515_24H:BRD-A28105619-001-01-3:10	Cucurbitacin-i	**−0.0531**	**2.51E-09**	**3.98E-06**	**1.81**	**−15.59**
CPC006_HCC515_6H:BRD-K16406336-311-01-2:10	Methylene-blue	**−0.0544**	**6.11E-09**	**8.44E-06**	**1.77**	**−14.52**
CPC016_MCF7_24H:BRD-K08547377-003-03-2:10	Irinotecan	**−0.0506**	**1.88E-08**	**2.12E-05**	**1.7**	**−13.16**
CPC001_VCAP_24H:BRD-K12516989-001-01-9:10	Zaprinast	**−0.0442**	**6.06E-08**	**5.64E-05**	**1.93**	**−13.91**
CPC016_NPC_24H:BRD-A22783572-065-01-3:10	Vinblastine	**−0.0493**	**1.28E-07**	**1.08E-04**	**1.69**	**−11.63**
CPC004_PC3_6H:BRD-A69815203-001-05-0:10	Cyclosporin-a	**−0.0455**	**1.90E-07**	**1.51E-04**	**1.84**	**−12.37**
CPC008_PC3_6H:BRD-K66037923-001-04-4:10	BRD-K66037923	**−0.048**	**1.96E-07**	**1.53E-04**	**1.76**	**−11.79**
MUC.CP003_MCF7_24H:BRD-K02407574-001-04-8:0.3704	Parbendazole	**−0.0468**	**2.45E-07**	**1.79E-04**	**1.63**	**−10.75**
**Similar Molecular Signatures**						
CPC013_SKB_24H:BRD-K61175124-001-01-0:10	BRD-K61175124	0.0556	2.63E-13	1.12E-08	−1.83	23.09
CPC016_SKB_24H:BRD-A06352508-001-02-9:10	SB-218078	0.0544	1.17E-12	1.67E-08	−1.87	22.28
CPC006_HT29_24H:BRD-A67788537-001-01-7:120	Salermide	0.0493	1.78E-12	1.91E-08	−1.85	21.75
CPC002_PC3_6H:BRD-A22684332-003-03-1:10	Procaterol	0.0582	3.07E-12	2.63E-08	−1.64	18.87
CPC007_HT29_24H:BRD-A09719808-001-02-3:10	BRD-A09719808	0.0506	8.82E-12	6.29E-08	−1.81	20.02
CPC019_VCAP_6H:BRD-K23282736-001-01-1:10	BRD-K23282736	0.0594	1.12E-11	6.83E-08	−1.78	19.53
CPC007_HT29_6H:BRD-A69470004-019-04-0:10	BRD-A69470004	0.0556	5.64E-11	3.02E-07	−1.69	17.3
CPC013_SKB_24H:BRD-K74623475-001-02-7:10	BRD-K74623475	0.048	1.67E-10	7.94E-07	−1.86	18.18
CPC006_A673_6H:BRD-K84924563-001-01-2:40	BRD-K84924563	0.0531	3.62E-10	1.29E-06	−1.68	15.85
CPC013_SKB_24H:BRD-K16541732-001-01-3:10	BRD-K16541732	0.0493	7.61E-10	1.92E-06	−1.81	16.48

**Table 2:** Top pathways and their associated *P* values are stated in the table.

Pathway name	Pathway Id	*P* value	*P* value (FDR)	*P* value (Bonferroni)
Influenza A	05164	**7.635e-7**	**6.389e-5**	**9.849e-5**
Viral protein interaction with cytokine and cytokine receptor	04061	**1.533e-6**	**6.389e-5**	**1.978e-4**
Measles	05162	**1.665e-6**	**6.389e-5**	**2.148e-4**
Epstein-Barr virus infection	05169	**2.019e-6**	**6.389e-5**	**2.605e-4**
IL-17 signaling pathway	04657	**2.476e-6**	**6.389e-5**	**3.194e-4**

The *P* value corresponding to the pathway was computed using only over-representation analysis.

**Figure 4.**
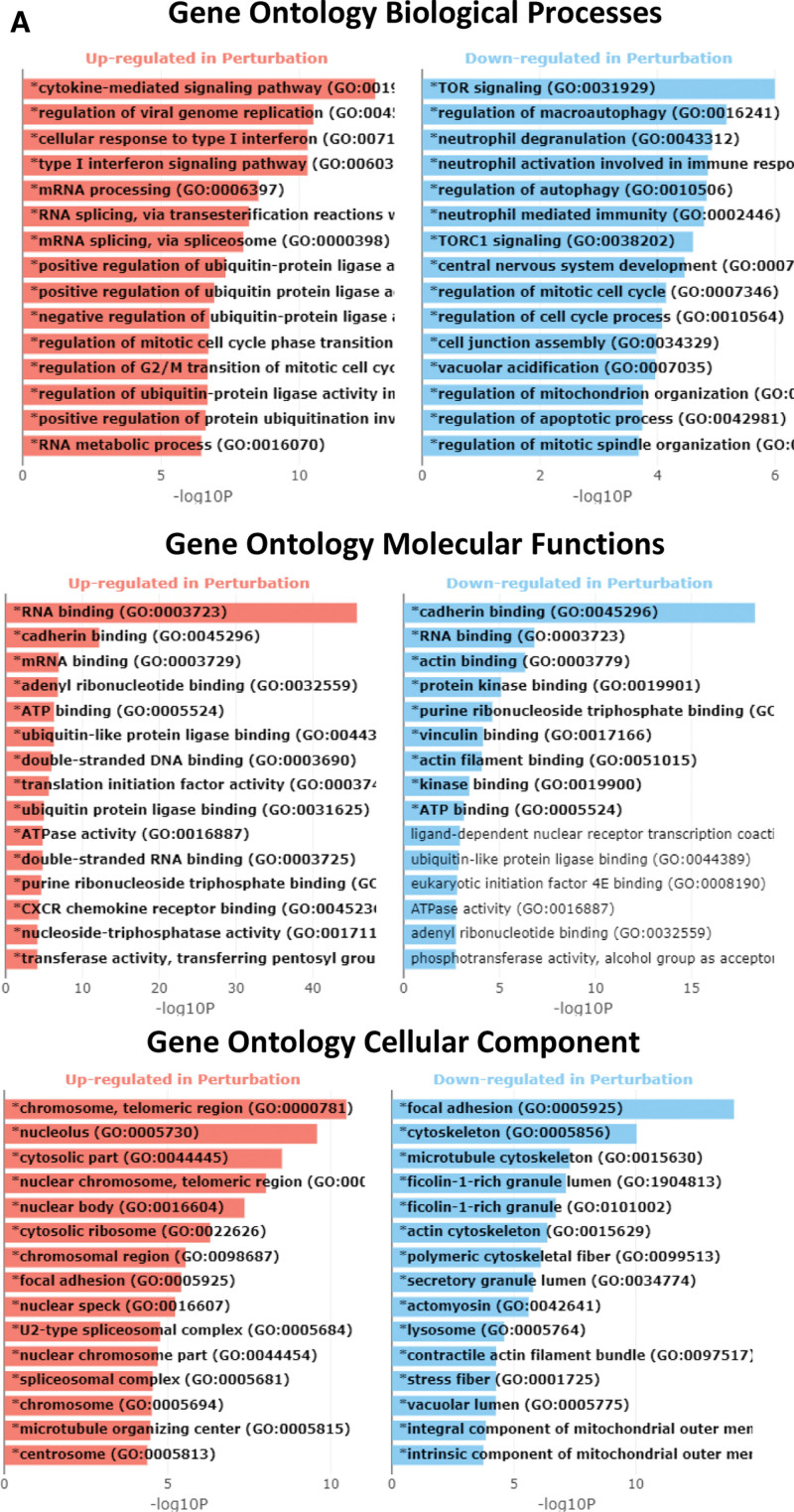
Gene Ontology Enrichment Analysis. The bar charts display the results of the Gene Ontology enrichment analysis generated using Enrichr. The x-axis indicates the −log10(*P* value) for each term. Significant terms are highlighted in bold.

**Figure 5.**
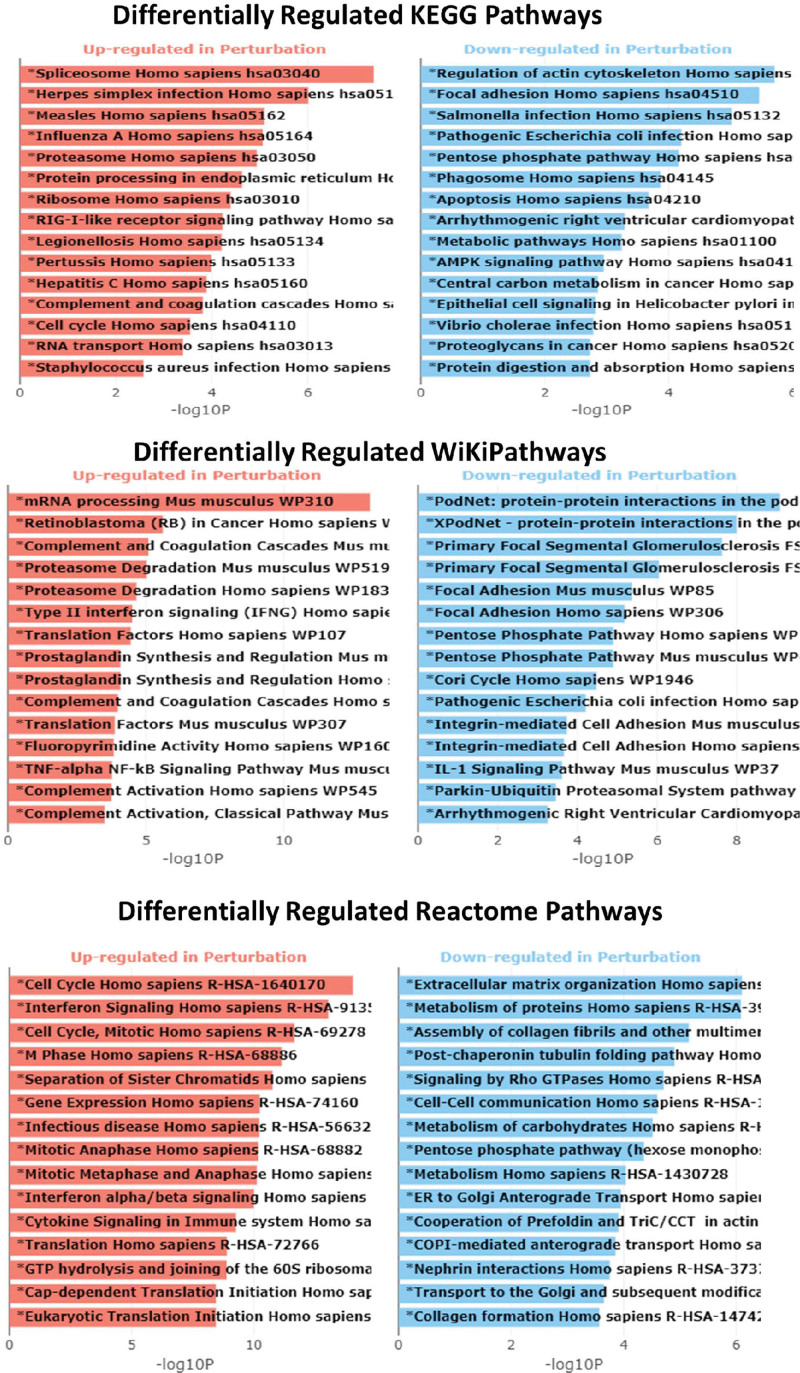
Pathway Enrichment Analysis. The bar charts displaying the results of the pathway enrichment analysis generated using Enrichr. The *x*-axis indicates the −log10(*P*-value) for each term. Significant terms are highlighted in bold.

The GO enrichment analysis for the biological processes, molecular function, and cellular components was generated using Enrichr (Fig. 4). The x-axis indicates the −log10(*P* value) for each term, and significant terms enriched in each GO category are highlighted in bold. Similarly, Figure 5 shows the results of pathway enrichment analysis using Enrichr. The x-axis indicates the −log10(*P* value) for each term, and the significantly enriched pathways (KEGG, Wiki pathways, and Reactome) are highlighted in bold.

In this experiment, 141 DEGs were identified from a total of 9665 DEGs obtained from BioJupies analysis of the RNASeq reads of SARS CoV-infected A549 cells and mock-treated A549 cells based on a *P* value cut-off (.05) and a fold change cut-off of 1.5. The DEGs were analyzed in the context of pathways obtained from the Kyoto Encyclopedia of Genes and Genomes (KEGG) database, gene ontologies from the Gene Ontology Consortium database, and iPathwayGuide analysis, which further showed that 34 pathways were significantly affected in the SARS CoV2 infected A549 cells compared to the mock-treated A549 cells. In addition, 557 Gene Ontology (GO) terms, 224 gene upstream regulators, 451 chemical upstream regulators, and 31 diseases were found to be significantly (*P* < .05) enriched before the correction for multiple comparisons.

The top five upstream regulators identified after the Bonferroni correction for signal transducer and activator of transcription 2 (STAT2), interferon regulatory factor 9 (IRF9), interferon-beta (IFNβ), interleukin-1-beta (IL-1β), and interferon regulatory factor 3 (IRF3) were predicted to be activated (Table 7).

**Table 7:** Top upstream regulators after Bonferroni Correction are given in the table.

Upstream Regulator (u)	DTA(u)	DT(u)	*P* value	*P* value (FDR)	*P* value (Bonferroni)
STAT2	11	11	**1.655e-14**	**6.848e-12**	**8.092e-12**
IRF9	10	10	**2.801e-14**	**6.848e-12**	**1.370e-11**
IFNB1	6	7	**1.526e-6**	**2.488e-4**	**7.464e-4**
IL1B	7	8	**1.188e-4**	**.014**	.058
IRF3	3	3	**1.464e-4**	**.014**	.072

COVID-19 infection activates key infectious disease-specific immune-related signaling pathways such as influenza A, viral protein interaction with cytokine and cytokine receptors, measles, Epstein-Barr virus infection, and IL-17 signaling pathway (Table 3). Likewise, significantly enriched Gene Ontology (GO) terms such as biological, molecular, and cellular processes based on the false discovery rate (*q* value) were identified using iPathwayGuide. The top identified biological processes were innate immune response, response to external biotic stimulus, response to other organisms, response to biotic stimulus, and defense response to other organisms, including chemokine receptor binding, chemokine activity, CXCR chemokine receptor binding, receptor-ligand activity, signaling receptor activator activity. The top cellular components identified included blood microparticles, fibrinogen complexes, nuclear outer membranes, extracellular spaces, and extracellular regions for each pruning type (Tables 4–6).

**Table 3:** Top identified biological processes. The top-scoring biological process, molecular function, and cellular component for each pruning type are described below in the table.

Pruning type: None				Pruning type: High-specificity		Pruning type: Smallest common denominator	
GO Term	*P* value	*P* value (FDR)	*P* value (Bonferroni)	GO Term	*P* value	GO Term	*P* value
**Biological processes**							
Innate immune response	1.000e-24	4.427e-22	4.427e-22	Type I interferon signaling pathway	2.380e-12	Type I interferon signaling pathway	9.961e-14
Response to external biotic stimulus	1.000e-24	6.549e-22	1.965e-21	Defense response to virus	8.301e-12	Defense response to virus	4.012e-13
Response to other organism	1.000e-24	6.549e-22	1.965e-21	Negative regulation of viral genome replication	2.490e-8	Interferon-gamma-mediated signaling pathway	1.383e-10
Response to biotic stimulus	3.000e-24	2.075e-21	8.301e-21	Interferon-gamma-mediated signaling pathway	8.301e-6	Negative regulation of viral genome replication	1.868e-8
Defense response to other organism	4.900e-23	2.712e-20	1.356e-19	Innate immune response			
**Molecular functions**							
Chemokine receptor binding	1.300e-9	3.897e-7	5.629e-7	Chemokine activity	7.794e-7	Chemokine receptor binding	5.629e-7
Chemokine activity	1.800e-9	3.897e-7	7.794e-7	CXCR chemokine receptor binding	4.546e-5	2′-5′-Oligoadenylate synthetase activity	.040
CXCR chemokine receptor binding	1.300e-8	1.876e-6	5.629e-6	2′-5′-Oligoadenylate synthetase activity	.030	Double-stranded RNA binding	.040
Receptor ligand activity	1.700e-6	1.840e-4	7.361e-4	Double-stranded RNA binding	.030	Protein ADP-ribosylase activity	.086
Signaling receptor activator activity	2.400e-6	2.078e-4	.001	Protein ADP-ribosylase activity	.068	ISG15 Transferase activity	.109
**Cellular components**							
Blood microparticle	9.800e-8	3.763e-5	3.763e-5	Blood microparticle	3.763e-5	Blood microparticle	3.763e-5
Fibrinogen complex	.002	.358	.768	Fibrinogen complex	.358	Fibrinogen complex	.358
Nuclear outer membrane	.003	.358	1.000	Nuclear outer membrane	.358	Nuclear outer membrane	.358
Extracellular space	.006	.425	1.000	Specific granule lumen	.553	Extracellular region	.553
Extracellular region	.008	.425	1.000	Costamere	.553	Specific granule lumen	.553

The upstream regulator drugs obtained either based on chemical synthesis or natural sources with opposite molecular signatures were also identified based on iPathwayGuide Analysis The drugs that can significantly reverse the molecular impact of COVID-19 infection are Methyl Prednisolone, Prednisolone, Gold Sodium Thiomalate, Tofacitinib, Diclofenac, JQ1 Compound, Azathioprine, etc. (Fig. 3B). The upstream regulator drugs and natural products with opposite molecular signatures identified using iPathwayGuide sorted based on the Z score are listed in Supplementary Table 1, http://links.lww.com/MD/G901.

In the present study, SwissTargetPrediction was performed for prednisolone and withaferin A, using the canonical SMILES code. The Open Targets Platform was applied to uncover Withaferin-A molecular targets associated with COVID-19 disease pathology. Scientific evidence was used in the Open Targets Platform to assign a score and rank target-disease associations and help target prioritization. Among the molecular targets of prednisolone and withaferin-a, 40 and 36 targets, respectively, were significantly associated with COVID-19 pathology (Table 8).

**Table 8:** COVID-19 associated targets regulated by prednisolone and withaferin-A.

Disease	Drug or natural product	Number of associated targets	Therapeutic area	All targets
COVID-19	Prednisolone	40	Infectious disease	DPP4 JAK1 NR3C1 JAK2 AR PTGS1 CHRNA4 IL6 PDE10A SLC5A2 FLT3 MAPK14 TYK2 OPRM1 KIT PPARG KDR ABL1 NR3C2 ESR2 CNR1 ADORA3 MPEG1 PGR ADAM17 CD38 MTOR MPO EGFR SLC6A3 MAPK1 ALK NOS2 SLC5A1 BRD4 MAPK3 ADK LCK RORA SHBG
COVID-19	Withaferin-A	36	Infectious disease	NR3C1 PTGS2 AR HMGCR PTGS1 GSK3B F10 PDE4D GSK3A IMPDH1 PDE3A PDE3B PDE10A MAPK14 JAK3 NR3C2 IKBKB ADORA2A PGR REN PARP1 ERBB2 CCR1 MAPK1 ALK HDAC3 PRKCB BRAF IL6ST CXCR3 MAPK8 IARS1 BRD4 BCL2L1 MAPK3 MDM2

## 4. Discussion

COVID-19 is highly infectious and pathogenic compared to other viral infections, and the exact mortality rate has yet to be determined because the pandemic is not yet under control in several countries.^[9,12]^ Therefore, deciphering the underlying pathologic mechanisms is central to identifying and developing COVID-19-specific drugs to effectively treat and prevent person-to-person transmission, COVID-19 complications, and reduce mortality. COVID-19 is usually characterized by cough, breathing problems, high body temperature, diarrhea, and abdominal discomfort, and in severe cases, it causes atypical pneumonia, SARS, stroke, thrombosis, multiple organ failure, and in some cases, death.^[3]^ It was found that approximately 80% of COVID-19 cases had mild or asymptomatic symptoms, with the elderly and those with other comorbid conditions more likely to develop severe symptoms and succumb to the disease.^[4,9]^

Distinguishing COVID-19 from other influenza viruses, SARS, and MERS coronaviruses is essential in the clinical setting to develop effective or efficient treatment strategies for patients.^[39]^ Noninfectious diseases such as idiopathic interstitial pneumonia, cryptogenic organizing pneumonia, dermatomyositis, and vasculitis also need to be differentially diagnosed from COVID-19^[7,9,39]^

The COVID -19 infection of A549 cells activated upstream genes, such as STAT2, IRF9, IFNB1, IL1B, and IRF3. Biological processes such as the type I interferon signaling pathway, defense response to viruses, negative regulation of viral genome replication, and interferon-gamma-mediated signaling pathways were differentially regulated. Molecular functions such as chemokine activity, CXCR chemokine receptor binding, 2′-5′-oligoadenylate synthetase activity, double-stranded RNA binding, and protein ADP-ribosylase activity were enriched in the COVID-infected cells. Cytokines are hormones of the immune system that are important for innate and adaptive host responses, cell growth and differentiation, repair and development, cellular homeostasis, and cell death.^[35,40,41]^ Cytokines are glycoproteins that are released upon any external stimulus and bind to specific cell surface receptors on the plasma membrane of target cells to elicit their responses.^[42–44]^

The cytokine/chemokine storm seen in moderate to severe cases of COVID -19 is caused by a significant increase in the levels of several circulating cytokines and chemokines, such as interleukin-6 (IL-6), interleukin-8 (IL-8), tumor necrosis factor-alpha (TNF-α), C-X-C motif chemokine ligand 10 (CXCL-10), and interferon-gamma induced protein-10 (IP-10), and contributes to poor prognosis.^[7,45]^ In general, viruses evolve mechanisms to avoid detection and subsequent destruction in the host by remodeling and copying cytokine and cytokine receptor genes.^[46,47]^ Similarly, COVID-19 induced cytokines, cytokine receptors, chemokines, and other specific cytokine receptors and binding proteins to destabilize and alter host cytokine responses and immune networks.^[16,45,47]^ Here, COVID-19-induced chemokines and cytokines can either enhance or prevent cytokine signaling and significantly alter or attenuate various arms of the host immunity. In addition, cellular processes such as the blood microparticle-fibrinogen complex were activated in COVID-infected A549 cells. The increase in cellular processes, such as blood microparticles, observed in the present study was confirmed by a recent study showing an increase in circulating blood microparticles and activated platelets in COVID-19 patients.^[48]^

The COVID-19 pandemic is currently being addressed with vaccines, convalescent plasma, monoclonal antibodies, antiviral drugs such as remdesivir, and preventive measures such as wearing masks, hand hygiene, and social distancing.^[49]^ In the present study, withaferin-A was predicted to counteract the molecular signatures triggered by COVID-19. Using NGKD platforms, we recently found that withaferin-A reverses the gene signatures induced by COVID-19 in NHBE cells.^[13]^

Analysis of the open-target platform revealed that 36 targets played a role in COVID-19 pathology. Withaferin-A is a constituent of the medicinal plant *W. somnifera* (Indian ginseng or ashwagandha). Its active constituents include withanolides, saponins, alkaloids, and steroidal lactones. *W. somnifera* is used in herbal preparations in traditional medicine and has antioxidant, anti-anxiety, anti-inflammatory, antibacterial, and aphrodisiac properties, among others^[50,51]^ Ashwagandha has neuroprotective, cardioprotective, immunomodulatory, and anticancer properties.^[51]^ In a recent in silico screening study, ashwagandha was also found to contain natural compounds against COVID-19.^[52]^

Traditional Chinese Medicine (TCM) has also been used in the treatment of COVID-19.^[53]^ The traditional Chinese herbal formula, JinFuKang, consists of 12 medicinal plants, with each dose containing 10 mL.^[54]^ JinFuKang has anticancer properties and numerous medicinal benefits.^[54]^ Antiviral remdesivir reduces mortality only very slightly,^[56]^ the use of corticosteroids increases the possibility of secondary infections,^[57]^ and monoclonal antibody therapies are either expensive or difficult to obtain for COVID-19 therapy. However, oral antiviral drugs such as paxlovid and molnupiravir introduced by Pfizer and Merck, respectively, are authorized by the Food and Drug Administration (FDA), USA, for COVID-19 treatment^.[58]^ Nevertheless, it may also be valuable to explore the gene signatures triggered by COVID-19 and its variants in different experimental model systems to identify potential drugs or natural products for COVID-19 therapy.

## 5. Conclusions

The present study demonstrated the application of RNA sequencing technologies in conjunction with NGKD platforms to decipher specific compounds, either synthetic or derived from natural products, for the potential amelioration of COVID-19. However, further in-depth studies are needed to validate drugs such as prednisolone, methylprednisolone, diclofenac, and JQ1, and natural products such as Withaferin-A and JinFuKang in COVID-19 infection model systems, such as primary human alveolar epithelial cells and human small intestinal organoids (hSIOs)^[1,2]^ to determine mechanisms of action before preclinical and clinical trials for the potential treatment of COVID-19 and related pathologies. In conclusion, this study outlines a valuable method for applying NGKD platforms to discover precise drugs and natural products for the potential treatment of COVID-19-related disease pathologies.

**Table 4:** Top upstream regulators after Bonferroni Correction is given in the table.

Upstream Regulator (u)	DTA(u)	DT(u)	*P* value	*P* value (FDR)	*P* value (Bonferroni)
STAT2	11	11	**1.655e-14**	**6.848e-12**	**8.092e-12**
IRF9	10	10	**2.801e-14**	**6.848e-12**	**1.370e-11**
IFNB1	6	7	**1.526e-6**	**2.488e-4**	**7.464e-4**
IL1B	7	8	**1.188e-4**	**.014**	.058
IRF3	3	3	**1.464e-4**	**.014**	.072

**Table 5:** Top identified molecular functions. Only the top scoring molecular function for each pruning type is described below the table.

Pruning type: None				Pruning type: High-specificity		Pruning type: Smallest common denominator	
GO term	*P* value	*P* value (FDR)	*P* value (Bonferroni)	GO Term	*P* value	GO Term	*P* value
chemokine receptor binding	**1.300e-9**	**3.897e-7**	**5.629e-7**	Chemokine activity	**7.794e-7**	Chemokine receptor binding	**5.629e-7**
chemokine activity	**1.800e-9**	**3.897e-7**	**7.794e-7**	CXCR chemokine receptor binding	**4.546e-5**	2′-5′-Oligoadenylate synthetase activity	**.040**
CXCR chemokine receptor binding	**1.300e-8**	**1.876e-6**	**5.629e-6**	2′-5′-Oligoadenylate synthetase activity	**.030**	Double-stranded RNA binding	**.040**
receptor ligand activity	**1.700e-6**	**1.840e-4**	**7.361e-4**	Double-stranded RNA binding	**.030**	Protein ADP-ribosylase activity	**.086**
signaling receptor activator activity	**2.400e-6**	**2.078e-4**	**.001**	Protein ADP-ribosylase activity	**.068**	ISG15 transferase activity	**.109**

**Table 6:** Top identified cellular components. Only the top scoring cellular component for each pruning type is described below the table.

Pruning type: None				Pruning type: High-specificity		Pruning type: Smallest common denominator	
GO Term	*P* value	*P* value (FDR)	*P* value (Bonferroni)	GO Term	*P* value	GO Term	*P* value
Blood microparticle	**9.800e-8**	**3.763e-5**	**3.763e-5**	Blood microparticle	**3.763e-5**	Blood microparticle	**3.763e-5**
Fibrinogen complex	**.002**	.358	.768	Fibrinogen complex	**.358**	Fibrinogen complex	**.358**
Nuclear outer membrane	**.003**	.358	1.000	Nuclear outer membrane	**.358**	Nuclear outer membrane	**.358**
Extracellular space	**.006**	.425	1.000	Specific granule lumen	**.553**	Extracellular region	**.553**
Extracellular region	**.008**	.425	1.000	Costamere	**.553**	Specific granule lumen	**.553**

## Acknowledgments

The authors gratefully acknowledge the technical and financial support from King Abdulaziz University, DSR, Jeddah. Saudi Arabia

## Author contributions

PNP, LAD, LD, SB, and MR designed experiments. PNP and MR conducted experiments. PNP, LAD, LD, SB, and MR analyzed the data. PNP and MR prepared the manuscript. PNP and MR revised the manuscript. All authors contributed to the editing of the manuscript and scientific discussions.

## Supplementary Material



## References

[1] Lamers MM Beumer J van der Vaart J. SARS-CoV-2 productively infects human gut enterocytes. Science. 2020;369:50–4. 32358202 10.1126/science.abc1669 PMC7199907

[2] Mulay A Konda B Garcia G. SARS-CoV-2 infection of primary human lung epithelium for COVID-19 modeling and drug discovery. Cell Rep. 2021;35:109055. 33905739 10.1016/j.celrep.2021.109055 PMC8043574

[3] Damiati LA Bahlas S Aljohaney A. Implications of SARS-CoV-2 infection on the clinical, hematological, and inflammatory parameters in COVID-19 patients: a retrospective cross-sectional study. J Infect Public Health. 2022;15:214–21. 35007842 10.1016/j.jiph.2021.12.013 PMC8734060

[4] Wang C Horby PW Hayden FG. A novel coronavirus outbreak of global health concern. Lancet. 2020;395:470–3. 31986257 10.1016/S0140-6736(20)30185-9 PMC7135038

[5] Wu F Zhao S Yu B. A new coronavirus associated with human respiratory disease in China. Nature. 2020;579:265–9. 32015508 10.1038/s41586-020-2008-3 PMC7094943

[6] Agarwal A Leisegang K Panner Selvam MK. An online educational model in andrology for student training in the art of scientific writing in the COVID-19 pandemic. Andrologia. 2021;53:e13961. 33491204 10.1111/and.13961 PMC7995002

[7] Liu J Zheng X Tong QX. Overlapping and discrete aspects of the pathology and pathogenesis of the emerging human pathogenic coronaviruses SARS-CoV, MERS-CoV, and 2019-nCoV. J Med Virol. 2020;92:491–4. 32056249 10.1002/jmv.25709 PMC7166760

[8] Dong E Du H Gardner L. An interactive web-based dashboard to track COVID-19 in real time. Lancet Infect Dis. 2020;20:533–4. 32087114 10.1016/S1473-3099(20)30120-1 PMC7159018

[9] Epidemiology Working Group for NCIP Epidemic Response, Chinese Center for Disease Control and Prevention. The epidemiological characteristics of an outbreak of 2019 novel coronavirus diseases (COVID-19) in China. Zhonghua Liu Xing Bing Xue Za Zhi. 2020;41:145–51 32064853 10.3760/cma.j.issn.0254-6450.2020.02.003

[10] Yang J Petitjean SJL Koehler M. Molecular interaction and inhibition of SARS-CoV-2 binding to the ACE2 receptor. Nat Commun. 2020;11:4541. 32917884 10.1038/s41467-020-18319-6 PMC7486399

[11] Bezbaruah R Borah P Kakoti BB. Developmental landscape of potential vaccine candidates based on viral vector for prophylaxis of COVID-19. Front Mol Biosci. 2021;8. 10.3389/fmolb.2021.635337 PMC8082173 33937326

[12] Mandolesi M Sheward DJ Hanke L. SARS-CoV-2 protein subunit vaccination of mice and rhesus macaques elicits potent and durable neutralizing antibody responses. Cell Rep Med. 2021;2. 10.1016/j.xcrm.2021.100252 PMC8020888 33842900

[13] Pushparaj PN Abdulkareem AA Naseer MI. Identification of novel gene signatures using next-generation sequencing data from COVID-19 infection models: focus on neuro-COVID and potential therapeutics. Front Pharmacol. 2021;12. 10.3389/fphar.2021.688227 PMC8438179 34531741

[14] Pushparaj PN Kalamegam G Wali Sait KH. Decoding the role of astrocytes in the entorhinal cortex in Alzheimer’s disease using high-dimensional single-nucleus RNA sequencing data and next-generation knowledge discovery methodologies: focus on drugs and natural product remedies for dementia. Front Pharmacol. 2022;12. 10.3389/fphar.2021.720170 PMC8918735 35295737

[15] Torre D Lachmann A Ma’ayan A. BioJupies: automated generation of interactive notebooks for RNA-seq data analysis in the cloud. Cell Syst. 2018;7:556–561.e3. 30447998 10.1016/j.cels.2018.10.007 PMC6265050

[16] Blanco-Melo D Nilsson-Payant BE Liu WC. Imbalanced host response to SARS-CoV-2 drives development of COVID-19. Cell. 2020;181:1036–45 e9. 32416070 10.1016/j.cell.2020.04.026 PMC7227586

[17] Ritchie ME Phipson B Wu D. limma powers differential expression analyses for RNA-sequencing and microarray studies. Nucleic Acids Res. 2015;43:e47. 25605792 10.1093/nar/gkv007 PMC4402510

[18] Fernandez NF Gundersen GW Rahman A. Clustergrammer, a web-based heatmap visualization and analysis tool for high-dimensional biological data. Sci Data. 2017;4:170151. 28994825 10.1038/sdata.2017.151 PMC5634325

[19] Kuleshov MV Jones MR Rouillard AD. Enrichr: a comprehensive gene set enrichment analysis web server 2016 update. Nucleic Acids Res. 2016;44:W90–7. 27141961 10.1093/nar/gkw377 PMC4987924

[20] Benjamini Y Hochberg Y. Controlling the false discovery rate: a practical and powerful approach to multiple testing. J Royal Stat Soc. 1995;57:289–300.

[21] Benjamini Y Yekutieli D. The control of the false discovery rate in multiple testing under dependency. The Annals of Statistics. 2001;29:1165–88

[22] Duan QN Reid S Clark NR. L1000CDS(2): LINCS L1000 characteristic direction signatures search engine. NPJ Syst Biol Appl. 2016;2. 10.1038/npjsba.2016.15 PMC5389891 28413689

[23] Wang ZC Lachmann A Keenan AB. 1000FWD: fireworks visualization of drug-induced transcriptomic signatures. Bioinformatics. 2018;34:2150–2. 29420694 10.1093/bioinformatics/bty060 PMC6454499

[24] Draghici S Khatri P Tarca AL. A systems biology approach for pathway level analysis. Genome Res. 2007;17:1537–45. 17785539 10.1101/gr.6202607 PMC1987343

[25] Bonferroni CE. Il calcolo delle assicurazioni su gruppi di teste. In: Studi in onore del professore salvatore ortu carboni (Rome: Tipografia del Senato). 1935;13–60.

[26] Bonferron CE. Teoria statistica delle classi e calcolo delle probabilita. In: Pubblicazioni del istituto superiore di scienze economiche e commerciali di firenze (Firenze: Seeber). 1936;8:3–62

[27] Kanehisa M Goto S Sato Y. Data, information, knowledge and principle: back to metabolism in KEGG. Nucleic Acids Res. 2014;42:D199–205. 24214961 10.1093/nar/gkt1076 PMC3965122

[28] Ashburner M Lewis S. On ontologies for biologists: the Gene Ontology--untangling the web. Novartis Found Symp. 2002;247:664244–809052 12539950

[29] Harris MA Clark J Ireland A. The Gene Ontology (GO) database and informatics resource. Nucleic Acids Res. 2004;32:D258–61. 14681407 10.1093/nar/gkh036 PMC308770

[30] Draghici S Khatri P Martins RP. Global functional profiling of gene expression. Genomics. 2003;81:98–104. 12620386 10.1016/s0888-7543(02)00021-6

[31] Draghici S Khatri P Bhavsar P. Onto-tools, the toolkit of the modern biologist: onto-express, onto-compare, onto-design and onto-translate. Nucleic Acids Res. 2003;31:3775–81. 12824416 10.1093/nar/gkg624 PMC169030

[32] Draghici S. Statistics and Data Analysis for Microarrays Using R and Bioconductor. 2nd Edn. London: Chapman and Hall/CRC, 2011.

[33] Fisher RA. Statistical Methods for Research Workers. 11th Edn. Edinburgh, UK: Oliver & Boyd, 1925.

[34] Daina A Zoete V. Application of the swissdrugdesign online resources in virtual screening. Int J Mol Sci . 2019;20. 10.3390/ijms20184612 PMC6770839 31540350

[35] Bahlas S Damiati LA Al-Hazmi AS. Decoding the role of sphingosine-1-phosphate in asthma and other respiratory system diseases using next generation knowledge discovery platforms coupled with Luminex multiple analyte profiling technology. Front Cell Dev Biol. 2020;8:444. 32637407 10.3389/fcell.2020.00444 PMC7317666

[36] Kalamegam G Alfakeeh SM Bahmaid AO. In vitro evaluation of the anti-inflammatory effects of thymoquinone in osteoarthritis and in silico analysis of inter-related pathways in age-related degenerative diseases. Front Cell Dev Biol. 2020;8:646. 32793594 10.3389/fcell.2020.00646 PMC7391788

[37] Koscielny G An P Carvalho-Silva D. Open Targets: a platform for therapeutic target identification and validation. Nucleic Acids Res. 2017;45:D985–94. 27899665 10.1093/nar/gkw1055 PMC5210543

[38] Carvalho-Silva D Pierleoni A Pignatelli M. Open Targets Platform: new developments and updates two years on. Nucleic Acids Res. 2019;47:D1056–65. 30462303 10.1093/nar/gky1133 PMC6324073

[39] Huang C Wang Y Li X. Clinical features of patients infected with 2019 novel coronavirus in Wuhan, China. Lancet. 2020;395:497–506. 31986264 10.1016/S0140-6736(20)30183-5 PMC7159299

[40] Jafri MA Kalamegam G Abbas M. Deciphering the association of cytokines, chemokines, and growth factors in chondrogenic differentiation of human bone marrow mesenchymal stem cells using an ex vivo osteochondral culture system. Front Cell Dev Biol. 2019;7:380. 32010693 10.3389/fcell.2019.00380 PMC6979484

[41] Harakeh S Kalamegam G Pushparaj PN. Chemokines and their association with body mass index among healthy Saudis. Saudi J Biol Sci. 2020;27:6–11. 31889810 10.1016/j.sjbs.2019.03.006 PMC6933256

[42] Arango Duque G Descoteaux A. Macrophage cytokines: involvement in immunity and infectious diseases. Front Immunol. 2014;5:491. 25339958 10.3389/fimmu.2014.00491 PMC4188125

[43] Pushparaj PN. “Multiple Analyte Profiling (xMAP) technology coupled with functional bioinformatics strategies: potential applications in protein biomarker profiling in autoimmune inflammatory diseases,” in Essentials of Bioinformatics. 2019;II:151–65

[44] Pushparaj PN. “Translational interest of immune profiling,” in Precision Medicine for Investigators, Practitioners and Providers. 2020;105–122.

[45] Vaninov N. In the eye of the COVID-19 cytokine storm. Nat Rev Immunol. 2020;20:277. 10.1038/s41577-020-0305-6 PMC7132547 32249847

[46] Mogensen TH Paludan R. Molecular pathways in virus-induced cytokine production. Microbiol Mol Biol Rev. 2001;65:131–50 11238989 10.1128/MMBR.65.1.131-150.2001 PMC99022

[47] Florindo HF Kleiner R Vaskovich-Koubi D. Immune-mediated approaches against COVID-19. Nat Nanotechnol. 2020;15:630–45. 32661375 10.1038/s41565-020-0732-3 PMC7355525

[48] Zahran AM El-Badawy O Ali WA. Circulating microparticles and activated platelets as novel prognostic biomarkers in COVID-19; relation to cancer. PLoS One. 2021;16:e0246806. 33617530 10.1371/journal.pone.0246806 PMC7899358

[49] Sewell HF Agius RM Kendrick D. Vaccines, convalescent plasma, and monoclonal antibodies for Covid-19. BMJ. 2020;370:m2722. 32646867 10.1136/bmj.m2722

[50] Sood A Mehrotra A Dhawan DK. Indian Ginseng (*Withania somnifera*) supplementation ameliorates oxidative stress and mitochondrial dysfunctions in experimental model of stroke. Metab Brain Dis. 2018;33:1261–74. 29671210 10.1007/s11011-018-0234-2

[51] Singh N Yadav SS Rao AS. Review on anticancerous therapeutic potential of *Withania somnifera* (L.) Dunal. J Ethnopharmacol. 2021;270:113704. 33359918 10.1016/j.jep.2020.113704

[52] Srivastava A Siddiqui S Ahmad R. Exploring nature’s bounty: identification of *Withania somnifera* as a promising source of therapeutic agents against COVID-19 by virtual screening and in silico evaluation. J Biomol Struct Dyn. 2022;40:1858–908. 33246398 10.1080/07391102.2020.1835725 PMC7755033

[53] Yang Y Islam MS Wang J. Traditional Chinese medicine in the treatment of patients infected with 2019-new Coronavirus (SARS-CoV-2): a review and perspective. Int J Biol Sci. 2020;16:1708–17. 32226288 10.7150/ijbs.45538 PMC7098036

[54] Cassileth BR Rizvi N Deng G. Safety and pharmacokinetic trial of docetaxel plus an Astragalus-based herbal formula for non-small cell lung cancer patients. Cancer Chemother Pharmacol. 2009;65:67–71. 19421753 10.1007/s00280-009-1003-z PMC3746541

[55] Que ZJ Yang Y Liu HT. Jinfukang regulates integrin/Src pathway and anoikis mediating circulating lung cancer cells migration. J Ethnopharmacol. 2021;267:113473. 33068649 10.1016/j.jep.2020.113473

[56] Wilt TJ Kaka AS MacDonald R. Remdesivir for adults with COVID-19: a living systematic review for American College of Physicians practice points. Ann Intern Med. 2021;174:209–20. 33017170 10.7326/M20-5752 PMC7564604

[57] Gopalaswamy R Subbian S. Corticosteroids for COVID-19 therapy: potential implications on tuberculosis. Int J Mol Sci . 2021;22. 10.3390/ijms22073773 PMC8038708 33917321

[58] Cully M. A tale of two antiviral targets - and the COVID-19 drugs that bind them. Nat Rev Drug Discov. 2022;2149:3–5. 10.1038/d41573-021-00202-8 34857884

