# Supplementary material for: Deciphering SARS CoV-2-associated pathways from RNA sequencing data of COVID-19-infected A549 cells and potential therapeutics using in silico methods
Source: Medicine (Baltimore). 2022 Sep 2;101(35):e29554. doi: 10.1097/MD.0000000000029554 (PMC9439635; doi:10.1097/MD.0000000000029554)
Supplement: Supplementary file 1 [file medi-101-e29554-s001.pdf]

Supplementary Table 1 The upstream regulator drugs and natural products with opposite molecular signatures identified using iPathwayGuide sorted based on Z score.

Chemical Name	cDE_n	cDE	cAll	pv_comb_n_fdr	pv_n_fdr	pv_zscore_fdr	zscore
Doxorubicin	63	79	4588	0.000769	1	0.000407	-4.71986
potassium chromate(VI)	43	51	2029	9.37E-06	0.137023	0.000407	-4.71495
Methylprednisolone	21	21	48	9.65E-14	7.41E-24	0.000446	-4.58258
Antirheumatic Agents	35	40	2332	0.001029	1	0.000446	-4.52904
(+)-JQ1 compound	52	63	1954	2.07E-08	0.0002	0.000446	-4.52027
Prednisolone	18	18	63	2.23E-13	1.18E-16	0.001225	-4.24264
dicrotophos	34	41	2782	0.005795	1	0.001225	-4.21669
Azathioprine	23	24	418	1.47E-09	3.72E-06	0.001225	-4.2
Gold Sodium Thiomalate	20	21	47	2.26E-13	1.31E-22	0.001423	-4.14614
jinfukang	45	59	2710	0.002421	0.672866	0.002113	-4.03586
Urethane	33	42	2850	0.037143	1	0.007165	-3.70328
perfluorooctane sulfonic acid	16	18	482	0.000733	0.073249	0.025713	-3.29983
tofacitinib	10	10	17	5.84E-12	1.01E-12	0.0371	-3.16228
Diclofenac	15	15	55	5.84E-12	2.06E-13	0.0371	-3.15296
Methotrexate	28	33	896	0.000138	0.004062	0.07071	-2.91999

Supplementary Table 02 COVID-19 Associated Targets Regulated by Prednisolone and Withaferin-A

**Disease**	**Drug or Natural Product**	**Number of associated targets**	**Therapeutic Area**	**All targets**
COVID-19	Prednisolone	40	infectious disease	DPP4 JAK1 NR3C1 JAK2 AR PTGS1 CHRNA4 IL6 PDE10A SLC5A2 FLT3 MAPK14 TYK2 OPRM1 KIT PPARG KDR ABL1 NR3C2 ESR2 CNR1 ADORA3 MPEG1 PGR ADAM17 CD38 MTOR MPO EGFR SLC6A3 MAPK1 ALK NOS2 SLC5A1 BRD4 MAPK3 ADK LCK RORA SHBG
COVID-19	Withaferin-A	36	infectious disease	NR3C1 PTGS2 AR HMGCR PTGS1 GSK3B F10 PDE4D GSK3A IMPDH1 PDE3A PDE3B PDE10A MAPK14 JAK3 NR3C2 IKBKB ADORA2A PGR REN PARP1 ERBB2 CCR1 MAPK1 ALK HDAC3 PRKCB BRAF IL6ST CXCR3 MAPK8 IARS1 BRD4 BCL2L1 MAPK3 MDM2
